# Axillary Brachial Plexus Block Compared with Other Regional Anesthesia Techniques in Distal Upper Limb Surgery: A Systematic Review and Meta-Analysis

**DOI:** 10.3390/jcm13113185

**Published:** 2024-05-29

**Authors:** Kristof Nijs, Pieter ‘s Hertogen, Simon Buelens, Marc Coppens, An Teunkens, Hassanin Jalil, Marc Van de Velde, Layth Al Tmimi, Björn Stessel

**Affiliations:** 1 Department of Anesthesiology and Pain Medicine, Jessa Hospital, 3500 Hasselt, Belgium; 2 Faculty of Medicine and Life Sciences, University of Hasselt, 3590 Diepenbeek, Belgium; 3 Department of Cardiovascular Sciences, University of Leuven, 3000 Leuven, Belgium; 4 Department of Anesthesiology and Pain Medicine, University Hospitals Leuven, 3000 Leuven, Belgium; 5 Department of Anesthesiology and Perioperative Medicine, University Hospital Ghent, 9000 Ghent, Belgium; 6 Department of Basic and Applied Medical Sciences, University Ghent, 9000 Ghent, Belgium

**Keywords:** axillary brachial plexus block, regional anesthesia, upper limb surgery

## Abstract

**Background:** Several regional anesthesia (RA) techniques have been described for distal upper limb surgery. However, the best approach in terms of RA block success rate and safety is not well recognized. **Objective:** To assess and compare the surgical anesthesia and efficacy of axillary brachial plexus block with other RA techniques for hand and wrist surgery. The attainment of adequate surgical anesthesia 30 min after block placement was considered a primary outcome measure. Additionally, successful block outcomes were required without the use of supplemental local anesthetic injection, systemic opioid analgesia, or the need to convert to general anesthesia. **Methods:** We performed a systematic search in the following databases: MEDLINE, EMBASE, Cochrane Database of Systematic Reviews, and CENTRAL. RCTs comparing axillary blocks with other brachial plexus block techniques, distal peripheral forearm nerve block, intravenous RA, and the wide-awake local anesthesia no tourniquet (WALANT) technique were included. **Results:** In total, 3070 records were reviewed, of which 28 met the inclusion criteria. The meta-analysis of adequate surgical anesthesia showed no significant difference between ultrasound-guided axillary block and supraclavicular block (RR: 0.94 [0.89, 1.00]; *p* = 0.06; I^2^ = 60.00%), but a statistically significant difference between ultrasound-guided axillary block and infraclavicular block (RR: 0.92 [0.88, 0.97]; *p* < 0.01; I^2^ = 53.00%). Ultrasound-guided infraclavicular blocks were performed faster than ultrasound-guided axillary blocks (SMD: 0.74 [0.30, 1.17]; *p* < 0.001; I^2^ = 85.00%). No differences in performance time between ultrasound-guided axillary and supraclavicular blocks were demonstrated. Additionally, adequate surgical anesthesia onset time was not significantly different between ultrasound-guided block approaches: ultrasound-guided axillary blocks versus ultrasound-guided supraclavicular blocks (SMD: 0.52 [−0.14, 1.17]; *p* = 0.12; I^2^ = 86.00%); ultrasound-guided axillary blocks versus ultrasound-guided infraclavicular blocks (SMD: 0.21 [−0.49, 0.91]; *p* = 0.55; I^2^ = 92.00%). **Conclusions:** The RA choice should be individualized depending on the patient, procedure, and operator-specific parameters. Compared to ultrasound-guided supraclavicular and infraclavicular block, ultrasound-guided axillary block may be preferred for patients with significant concerns of block-related side effects/complications. High heterogeneity between studies shows the need for more robust RCTs.

## 1. Introduction

Nowadays, surgery on the distal upper limb is generally performed in an ambulatory setting under regional anesthesia (RA), intravenous regional anesthesia (IVRA), or local anesthesia [1,2].

Avoiding general anesthesia (GA) is increasingly being recognized as the most suitable option to provide anesthesia for ambulatory hand and wrist surgery due to increased safety, reduced post-anesthesia care unit (PACU) length of stay, increased patient satisfaction, lower risk of postoperative nausea and vomiting, and numerous other benefits [3,4,5]. In the literature, rebound pain after the effect of RA has subsided has been described; however, recent studies comparing RA and GA did not prove this concept, and RA groups required fewer opioids [6,7]. Currently, the technique of choice for hand and wrist procedures may not be determined by evidence but often is based on the exact location of surgery, local institutional preferences, and the availability of drugs and equipment [2,8,9].

Multiple RA techniques for blocking the brachial plexus have been used to provide adequate anesthesia to the distal upper limb [2]. Most of these techniques rely on blocking the brachial plexus at different levels, such as the supraclavicular brachial plexus block (SCB), which targets the nerve structures at the level of the trunks. The infraclavicular brachial plexus (ICB) is at the cords and the axillary brachial plexus block (ABPB) is at the distinct nerves. Blocking the brachial plexus can be guided by ultrasound (US) or nerve stimulator (NS) or based on the landmark technique. Alternative RA techniques are IVRA (Bier’s block) or selective nerve blocks mid-humeral (MH) at the level of the forearm, either US-guided, NS-guided, or based on landmark techniques [8,9].

Depending on the type of surgery, one might prefer a different RA approach. For example, the selective blockade of peripheral nerves distally can be selected in specific types of hand surgery where motor sparing is essential when it comes to distal upper limb surgery, i.e., from the elbow down. Except for the forearm (FA) block and mini-Bier’s block, all aforementioned techniques theoretically cover the desired dermatomes, myotomes, and osteotomes.

ABPB has been shown to be a relatively easy block to perform [10], making it a frequently used nerve block for forearm and hand surgery [11]. After reviewing ABPB compared to other RA blocks, we performed a focused meta-analysis to evaluate the surgical anesthesia and efficacy of the US-guided ABPB compared to other US-guided approaches in providing RA for hand and wrist surgery. We hypothesized that ABPB is more efficacious in achieving a high-quality surgical block, has a fast onset time, and has a slightly better safety profile than other techniques.

## 2. Methods

This systematic review and meta-analysis was registered at PROSPERO (International Register of Prospective Reviews) [CRD42021255707]. Protocol development followed the PICOS (problem, intervention, comparator, outcome, and setting question) system. The review was reported based on the Preferred Reporting Items for Systematic Reviews and Meta-analyses (PRISMA) guideline for systematic reviews [12,13].

### 2.1. Search Strategy

We systematically searched the following databases: MEDLINE, EMBASE, Cochrane Database of Systematic Reviews, and CENTRAL between 1 January 1990 and 25 November 2022. Controlled vocabularies like medical subject headings (MeSH) and keyword terms were used to identify relevant articles. Search topic components per database are included in Appendix S1. The results were limited to English, Spanish, French, German, and Dutch languages, human studies, and adult patients (age > 18 years). We also checked clinical trial registries: US registry (www.clinicaltrials.gov), EU registry (www.clinicaltrialsregister.eu), and ISRCTN registry (WHO and ICMJE) (www.isrctn.com). Our last search took place on the 25 November 2022. We contacted the corresponding authors of identified trials for more information, especially regarding unpublished data.

### 2.2. Eligibility Criteria

We included studies performed in adult patients (age > 18 years) undergoing hand or wrist surgery under RA. Only randomized controlled trials (RCTs), regardless of blinding, that compared ABPB with other techniques of brachial plexus blockade such as interscalene block, SCB, ICB, mid-humeral block, or coracoid block were eligible. Furthermore, RCTs comparing ABPB with distal peripheral forearm nerve block, intravenous regional anesthesia (IVRA), or wide-awake local anesthesia no tourniquet (WALANT) were also included. Studies with combined regional and general anesthesia (GA) were excluded. Likewise, studies focusing on chronic pain were not included.

### 2.3. Study Selection and Reliability

Two independent reviewers (KN and SB) were responsible for the study selection. Studies were included for analysis based on predefined selection criteria. If any disagreement occurred between the two authors, a third author (BS) was consulted to make a final decision.

### 2.4. Data Extraction and Quality Assessment

**Figure 1:**
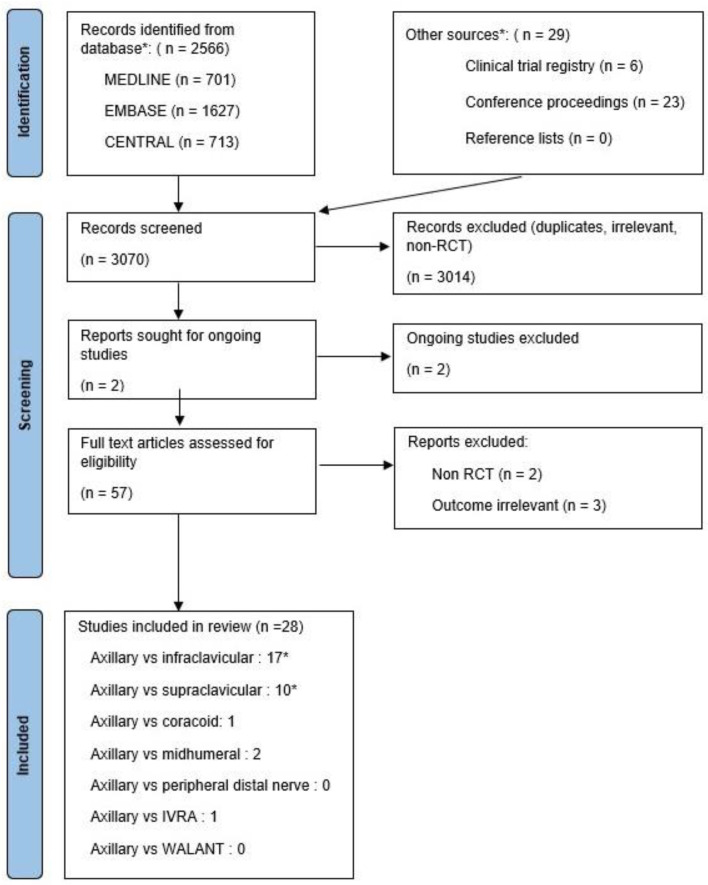
PRISMA diagram. * Three studies studied both supraclavicular and infraclavicular blocks. IVRA: intravenous regional anesthesia, WALANT: wide-awake local anesthesia no tourniquet.

The two reviewers independently screened article titles obtained by the previously defined search terms. Abstracts of potentially relevant articles were subsequently assessed. Abstracts of no apparent relevance were eliminated. Full-text manuscripts of all remaining studies were obtained, read, and qualitatively evaluated to result in the final article selection, as shown in Figure 1 below. Two authors (KN and SB) assessed the risk of bias in the trials and the quality of collected studies using the second version of the Cochrane risk-of-bias tool version 2 (ROB2) for randomized trials [14]. Using this standardized rating by the Cochrane group, the studies included in this review were evaluated on a fixed set of domains of bias, focusing on different aspects of trial design, conduct, and reporting, as presented in the Supplementary Document Appendices S2 and S3.

### 2.5. Outcome Measures

Primary outcome:

(1) Adequate surgical anesthesia 30 min after block completion and without needing supplemental local anesthesia (LA) injection, systemic analgesia (opioids), or general anesthesia (GA).

Secondary outcomes:

(2) The need for supplemental LA infiltration, additional RA block or systemic analgesia, or a combination to achieve adequate surgical anesthesia.
(3) The need for GA to achieve adequate surgical anesthesia.
(4) Performance time of RA block placement in minutes. No strict definition or method was specified in advance.
(5) Onset time of adequate surgical anesthesia. This was defined as the time in minutes from block completion to the absence or decrease of any sensation in the operative area where surgery would be conducted.
(6) Pain associated with RA block placement. No strict definition or method of assessment was specified in advance.
(7) Patient satisfaction. No strict definition or method of assessment was specified in advance.
(8) Block-related complications. Five complications were assessed: pneumothorax, vascular puncture, Horner’s syndrome, local anesthetic systemic toxicity (LAST), and neurological deficits, including residual neuropraxias unrelated to the surgical site, lasting more than 24 h. No strict definition or method of assessment was specified in advance. Some studies use trans-arterial RA guiding; these studies were excluded from evaluating block-related complications.
(9) Tourniquet pain. No strict definition or method of assessment was specified in advance.

### 2.6. Statistics

Statistics were performed using Revman (version 5.4). For the primary outcome, adequate surgical anesthesia, a meta-analysis was performed using the risk ratio as an effect size estimate. The Mantel–Haenszel model was fitted with fixed effects selected. We reviewed all RA techniques (NS-guided and US-guided), but only included ultrasound-based techniques in our meta-analysis, as nerve stimulation-based practices are falling out of favor, and the outcomes would depend on the technique. Trans-arterial approaches were excluded from the analysis for block-related complications. Only one RCT on the topic of IVRA was available. Therefore, no meta-analysis could be performed [1]. A meta-analysis was performed for the secondary outcomes: performance and onset time measures. Onset time was split into two groups (fast-onset LA mixtures and slow-onset LA mixtures). The meta-analysis was performed using an inverse variance model. Cohens D test, denoted in the forest plots as the standardized mean difference (SMD), was used to estimate the effect size and standard error. *p*-values < 0.05 were considered statistically significantly different.

## 3. Results

The full text was reviewed for 57 trials out of 3070 records, of which 28 RCTs with 6166 patients met the inclusion criteria. Figure 1 demonstrates the review process. The risk of bias was low for 11 studies, intermediate for 8, and high for 9 studies (Appendix S2). In 27 studies, ABPB (US- or NS-guided) and one trans-arterial ABPB were compared with another technique: ICB (US- or NS-guided) in 17 studies and SCB in 11 studies (US-guided or NS-guided). One study compared ABPB with IVRA.

Study characteristics and RA techniques are summarized in Table 1 and Appendix S4. All articles were RCTs with level I evidence. Three studies compared US-guided axillary, supraclavicular, and infraclavicular blocks [15,16,17]. LA medication and volumes are summarized in Appendix S5. A short-acting LA was used in eleven studies (lidocaine [15,18,19,20,21,22], mepivacaine [1,23,24,25,26]), a long-acting LA in eleven studies (bupivacaine [16,27,28,29,30], ropivacaine [17,31,32,33,34,35]), and a mixture of short- and long-acting LA in six studies [36,37,38,39,40,41]. Volumes and dosage of LA of the ABPB varied widely, with the lowest being 20 mL and the highest being 60 mL.

### 3.1. Primary Outcome: Adequate Surgical Anesthesia within 30 min of Block Completion

Out of the 28 studies, 25 studies reported on surgical anesthesia [1,16,17,18,19,20,21,22,23,24,25,26,27,28,29,30,31,32,33,34,35,36,37,40]. Only 17 studies involving 1254 participants conducted an evaluation for adequate surgical anesthesia at an interval of 30 min after block completion [16,18,19,20,22,23,25,27,28,30,31,33,34,35,36,37,40].

**Figure 2:**
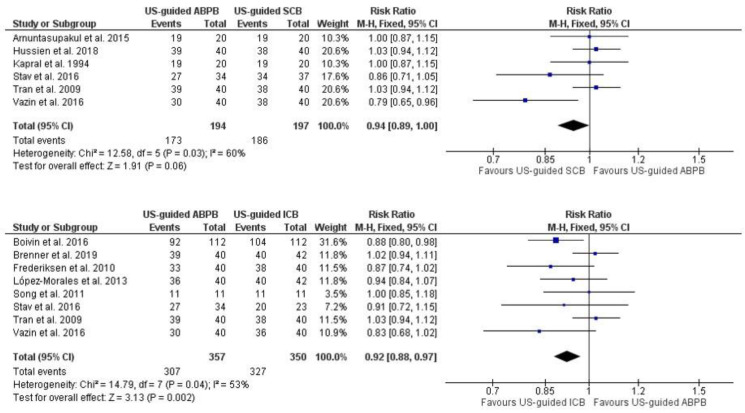
Forest plot of adequate surgical anesthesia at 30 min. US-guided: ultrasound-guided, ABPB: axillary brachial plexus block, ICB: infraclavicular block [15,16,17,18,19,20,23,27,29,30,36].

Pooled analysis of adequate surgical anesthesia within 30 min of block completion (no need for rescue analgesia) was not significantly different between US-guided APBP and US-guided SCB (RR: 0.94 [0.89, 1.00]; *p* = 0.06; I^2^ = 60.00%) (Figure 2). The meta-analysis of US-guided ABPB showed a statistically significant lower rate of adequate surgical anesthesia within 30 min compared to US-guided ICB (RR: 0.92 [0.88, 0.97]; *p* < 0.01; I^2^ = 53.00%) (Figure 2). One study compared US-guided ABPB and IVRA and showed no difference concerning adequate surgical anesthesia (not available (NA), *p* = 0.72) [1].

Two studies showed no differences in block adequacy for NS-guided APBP, NS-guided SCB, and a combined technique (ABPB + SCB combined) [21,26]. Five studies showed NS-guided ABPB to be equally as effective as NS-guided ICB [25,28,31,37,40]. One study showed trans-arterial ABPB to be similarly effective as US-guided ICB [24]. NS-guided ABPB more often resulted in adequate anesthesia compared to NS-guided ICB (83% vs. 53%, *p* = 0.03), as demonstrated by one study [35] and compared to NS-guided MHB (54% vs. 88%, *p* < 0.01) in another study [22].

### 3.2. The Need for Supplemental LA Infiltration, Additional RA Block or Systemic Analgesia, or a Combination to Achieve Adequate Surgical Anesthesia

Seventeen studies evaluated the need for supplemental anesthesia [1,15,16,17,18,21,22,23,24,25,27,31,34,35,36,37,40]. Two studies resorted to GA in the first instance and were excluded from the analysis [28,32]. One study combined all blocks with GA after block evaluation and found no significant difference in success rates (91% vs. 89%, NA) between ABPB and ICB [39].

The need for supplemental anesthesia to achieve adequate surgical anesthesia was found to be similar in US-guided ABPB compared to US-guided SCB, US-guided ICB, and IVRA in two, six, and one studies, respectively [1,15,16,18,23,24,27,36].

No different supplementation need was found between NS-guided ABPB and NS-guided ICB in three studies [25,37,40]. Also, between trans-arterial ABPB and US-guided ICB, no higher supplementation need was seen [24]. In NS-guided ABPB, the need for supplementation was not different compared to NS-guided CB [35]. Between NS-guided ABPB and NS-guided MHB, one study showed a significant difference in the need for LA supplementation (Bouaziz: 21% vs. 3%, *p* < 0.05) [22]; the other did not [34].

### 3.3. The Need for GA to Achieve Adequate Surgical Anesthesia

The need for GA for the completion of surgery to achieve adequate surgical anesthesia was reported in fifteen studies [1,16,18,19,22,23,24,25,27,28,31,32,34,36,40]. In those, no difference in the need for GA in ABPB compared to other techniques was found.

### 3.4. Performance Time of RA Block Placement in Minutes

Eighteen studies measured single-shot RA block performance time [16,17,18,19,20,22,23,24,27,30,31,32,34,35,36,37,38,39]. Two studies measured time-to-place catheter performance time and were excluded from the analysis [15,28].

**Figure 3:**
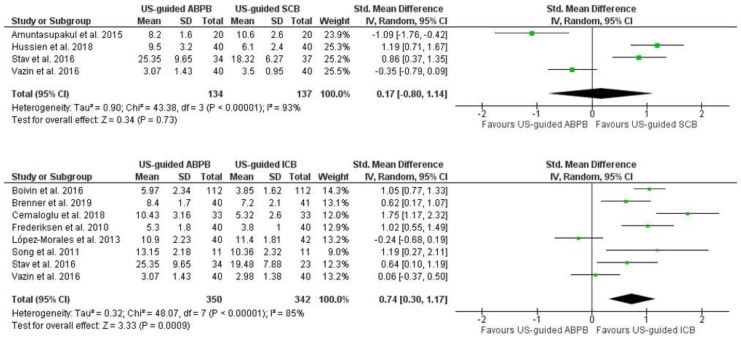
Forest plot of block performance time in minutes. US-guided: ultrasound-guided, NS-guided: nerve stimulation-guided, ABPB: axillary brachial plexus block, ICB: infraclavicular block, SCB: supraclavicular block [16,17,18,19,20,23,27,30,36,38].

Pooled analysis of performance time of block placement showed no significant difference between US-guided ABPB and US-guided SCB (Std MD = 0.17 min, 95% CI [−0.80, 1.14], *p* = 0.73, I^2^ = 93.00%) (Figure 3). The meta-analysis of US-guided ICB showed it to be performed significantly faster than US-guided ABPB (MD = 0.74 min, 95% CI [0.30, 1.17], *p* < 0.001, I^2^ = 85.00%) (Figure 3).

Between NS-guided ABPB and US-guided ICB, one study found a significantly longer performance time for ABPB (8.03 ± 3.92 min vs. 3.90 ± 2.27 min, *p* < 0.001) [39]. The meta-analysis of NS-guided ICB performance time showed it to be non-significantly different from NS-guided ABPB (MD = −0.56, 95% CI [−1.46, 2.58], *p* < 0.001, I^2^ = 0.59%) (Appendix S6). Between trans-arterial ABPB and US-guided ICB, a non-significant difference in performance time was found in one study (7 ± (SD 4) min vs. 7 ± (SD 3) min, *p* = 0.35) [24]. Koscielniak-Nielsen et al. found a non-significant difference in performance time between NS-guided ABPB and NS-guided CB (12 min vs. 11 min, NS) [35]. Between NS-guided ABPB and NS-guided MHB, Fuzier et al. showed a significantly longer performance time for MHB (8 min ± 3 vs. 13 min ± 5, *p* < 0.01) [34], while Bouaziz et al. found no difference (6 min ± 4 vs. 6 min ± 2, NS) [22].

### 3.5. Onset Time of Adequate Surgical Anesthesia

Sixteen studies reported outcomes on RA block onset time [17,20,21,22,23,24,27,30,31,33,34,35,37,39,40,41]. However, two studies did not precisely define this [37,41]. In three studies, block onset time was defined as the time from block completion to the onset of analgesia (and not anesthesia) [18,19,36].

**Figure 4:**
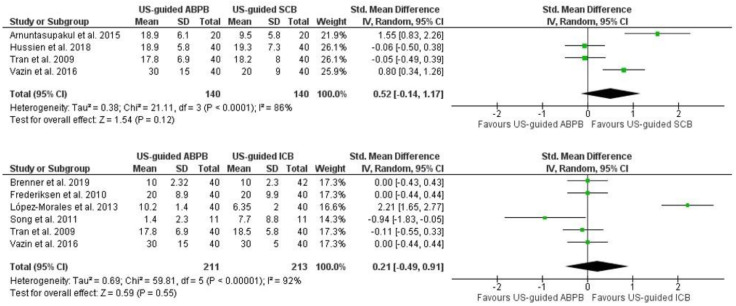
Forest plot of onset time in minutes. US-guided: ultrasound-guided, ABPB: axillary brachial plexus block, ICB: infraclavicular block, SCB: supraclavicular block [15,17,18,19,20,27,30,36].

The meta-analysis of the onset time of adequate surgical anesthesia showed no significant difference between US-guided ABPB and US-guided SCB (Std MD = 0.52 min, 95% CI [−0.14, 1.17], *p* = 0.12, I^2^ = 86.00%) (Figure 4). Pooled analysis of onset time for US-guided ABPB versus US-guided ICB showed no difference (Std MD = 0.21 min, 95% CI [−0.49, 0.91], *p* = 0.55, I^2^ = 92.00%) (Figure 4).

NS-guided ABPB showed a faster onset time compared to US-guided ICB in one study [39] and a similar onset time compared to NS-guided ICB in three studies [31,37,40]. In one study, the onset time for NS-guided ABPB was significantly longer versus NS-guided SCB (16.18 ± 2.65 min vs. 7.91 ± 2.29 min, *p* < 0.05) [21]. Dardón et al. did not specify regarding technique (US-guided or NS-guided); however, they reported a shorter “latency time” for ABPB (5 ± 2.3 min vs. 6 ± 1.5 min, *p* < 0.05) [41]. NS-guided ABPB showed a shorter onset time than NS-guided ICB in one study (Koscielniak-Nielsen 2000: 17 min vs. 30 min, *p* < 0.05) [35]. The onset time for NS-guided ABPB was shorter than that for NS-guided MHB according to Bouaziz et al. (15 ± 10 min vs. 25 ± 8 min, *p* < 0.05) [22], while there was no difference for Fuzier et al. (14 ± 6 min vs. 15 ± 6 min, NS) [34].

### 3.6. Pain Associated with RA Block Placement

Ten studies measured RA block performance-associated pain scores [15,17,20,23,30,34,35,36,37,38,39]. One study reported pain during RA block performance; however, this was not further defined [25].

Four studies found similar block-related pain scores between US-guided ABPB and US-guided SCB [17,20,24,30]. One study found that performing a US-guided ABPB was less comfortable compared to a US-guided ICB (VAS 3.2 ± 2.2 vs. 1.7 ± 1.9, *p* < 0.01) [38]. However, four studies showed similar block-related pain scores [17,23,24,36].

Tran et al. (2008) studied NS-guided ABPB and US-guided ICB and showed a higher block-related pain score for ABPB (4.17 ± 2.57 vs. 2.70 ± 2.02 on a 0–10 scale, *p* = 0.01) [39]. Two studies showed performing NS-guided ABPB to be less comfortable compared to NS-guided ICB [25,37]. NS-guided ABPB resulted in less block-related pain than NS-guided CB (VAS 0.6 (0–4) vs. 1.4 (0.1–3.8), *p* < 0.05) [35]. For NS-guided ABPB, a significantly lower block-related pain was found than for NS-guided MHB (verbal rating scale (VRS) 1–4: VRS of 1: 25 (56%) vs. 20 (44%), *p* < 0.05) [34].

### 3.7. Patient Satisfaction

Seven studies measured patient satisfaction after RA [1,19,23,27,32,37,38]. Four studies showed that patient satisfaction was similar for US-guided ABPB versus US-guided ICB [19,23,27,38]. One study on US-guided ABPB and IVRA showed that there was no difference in patient satisfaction except in young and healthy men [1]. Patients were asked if they would choose the same technique for future intervention and a non-significant difference was found (95% vs. 96.67%, *p* = 1.0). [1].

One study showed that patients were equally satisfied after NS-guided ABPB and NS-guided ICB [32,37].

### 3.8. Block-Related Complications

Twenty-one studies looked at (some) side effects of block performance [1,15,16,17,19,20,21,23,24,25,26,27,29,30,31,32,35,36,37,39,40].

Complications are summarized in Appendix S7. In the studies investigating US-guided ABPB, the presence of pneumothorax was found in only one study in the US-guided SCB group (95% CI [0.01, 2.00], *p* = 0.14, I^2^ = not applicable) (Appendix S8) [30]. Meta-analysis of the incidence of vascular puncture showed non-significantly different complication rates for US-guided ABPB compared to other US-guided blocks (95% CI [0.39, 1.89], *p* = 0.71, I^2^ = 0.00%) (Appendix S9). An analysis of Horner’s syndrome incidence showed it to be significantly less present in US-guided ABPB than in US-guided SCB (95% CI [0.01, 0.32], *p* = 0.002, I^2^ = 0.00%) (Appendix S10). A meta-analysis between US-guided ABPB and US-guided ICB was not possible because of only one study with Horner’s syndrome events.

Mild symptoms of LAST were reported in two studies, namely, Tran 2008: 2.86% of mild LAST (not further defined) in both NS-guided ABPB and US-guided ICB and Teunkens 2020: 3.3% reported incidence of tinnitus after cuff deflation in IVRA group, which was considered as a potential minor symptom of LAST [1,39].

### 3.9. Tourniquet Pain

Pain or discomfort related to the application of a surgical tourniquet on the upper arm was reported as an outcome in six studies. All these studies found no differences in pain or discomfort related to the application of a surgical tourniquet on the upper arm between ABPB and other techniques [1,19,31,34,35,36].

## 4. Discussion

This systematic review included a total of 28 studies comparing ABPB using different methods of targeting the brachial plexus (supraclavicular, infraclavicular, coracoid, mid-humeral) and IVRA [1,15,16,17,18,19,20,21,22,23,24,25,26,27,28,29,30,31,32,33,34,35,36,37,38,39,40,41]. No studies regarding distal peripheral nerve block, forearm IVRA, or WALANT were found.

The meta-analysis of adequate surgical anesthesia at 30 min showed no significant difference between US-guided ABPB and US-guided SCB; however, it did show a significant difference between US-guided ABPB and US-guided ICB. The absolute risk of block failure was low across all techniques, which means that the RR of 0.92 between ABPB and ICB is debatably clinically relevant. This is further corroborated by the data on secondary outcomes like the number of conversions to general anesthesia and supplemental local anesthetic infiltration not being significantly different. There was little difference between NS-guided and US-guided techniques regarding success rate. A well-known difficulty for ABPB is the musculocutaneous nerve, which has been observed to exhibit variability in shape, position, and echogenicity in the axillary fossa [42]. This may pose technical challenges, especially for those less experienced with axillary blocks, with the possibility of misidentification of the MCN and MCN block failure [42]. Also, we should interpret these results with caution as there is high heterogeneity between the studies.

This is corroborated by secondary outcome data showing similar need rates for supplemental LA infiltration, additional RA block, systemic analgesia, or a combination. No difference in the need for GA was found. Performance time was found to be shorter for ICB compared to ABPB. However, this performance time depends on the block’s guidance technique (US-guided, NS-guided, or trans-arterial). The onset time of the US-guided blocks was similar between the techniques. In practice, adding 5 min of performance time might not have an impact on operation room organization if there is a possibility of performing the block in tandem with a preceding procedure. If this is not possible and blocks are carried out sequentially, this might impact a busy operative list.

Pain associated with RA block placement was shown to be almost identical between ICB and ABPB. The use of NS guidance generated more patient discomfort than US-guided blocks, regardless of the block type. Patient satisfaction was rarely investigated in the included studies and showed similar satisfaction between ABPB, ICB, and IVRA. Variations in sedation (levels) during RA placement could have had an impact on procedural pain perception and patient satisfaction.

No serious adverse events were explicitly reported related to ABPB as the risks of pneumothorax, Horner’s syndrome, and transient phrenic nerve paralysis with ABPB were absent due to the technique [43].

The meta-analysis of the incidence of vascular puncture showed US-guided ABPB to be non-significantly better than the other US-guided blocks. Studies reporting on NS-guided ABPB and other NS-guided techniques all had higher incidences of vascular puncture. But, even within the NS-guided ABPB group, observed puncture rates differed substantially (0–30%), as can be seen in Addendum 7 [26,31,35,40].

The reported rate of pneumothorax was meager in all studies included in this review; only Hussien et al. reported an incidence of 10% in US-guided SCB [30]. However, potential underreporting of pneumothorax was possible due to underdiagnosis in the case of minor or absent symptoms. Although US guidance shows a reduction in the risk of pneumothorax, this theoretical risk should be taken into consideration when treating patients with a precarious respiratory status where ABPB or more distal techniques might be preferred [44].

The meta-analysis of the incidence of Horner’s syndrome showed US-guided ABPB to be significantly safer compared to other blocks. Of note, Horner’s syndrome, after RA, results from paralysis of the ipsilateral sympathetic cervical chain (stellate ganglion) and has a specific triad (ptosis, miosis, and exophthalmia) [45]. It is mainly associated with interscalene blocks and SCB blocks. However, it also has been described after US-guided ICB, although at a lower incidence, and can give patients discomfort, especially after ambulatory care [24,39,45,46]. Transient phrenic nerve paralysis was poorly described in the reviewed RCTs and probably underreported. It has been shown to be absent in ABPB; however, it has been described in the literature as up to 50% for SCB and 25% for ICB [43]. Potentially decreasing respiratory patient comfort and being a potential factor for unexpected hospital admission after ambulatory surgery.

LAST was only explicitly mentioned in two studies. Tran et al. noted the absence of any symptoms of LAST in US-guided ABPB and US-guided ICB, while Teunkens et al. reported tinnitus as a possible symptom of LAST when using the IVRA technique [1,15]. Teunkens et al. demonstrated 3.3% tinnitus after cuff deflation, which could indicate minor toxicity [1]. For IVRA, it is advised to keep the cuff inflated for at least 20 to 30 min after injection of the local anesthetic (LA) [9,47]. This also implies that for procedures shorter than 20 to 30 min, you might need to defer releasing the tourniquet until sufficient time has passed. The use of the forearm IVRA, which employs lower LA dosages with nearly identical anesthetic results and a lower risk of LAST, is another option for superficial hand and wrist surgery [9,48].

Tourniquet pain was found to be non-significantly different between techniques in all five studies reporting this phenomenon. The pathophysiology of tourniquet pain is incompletely understood [19]. The radial, musculocutaneous, medial cutaneous brachial (MCBN), and intercostobrachial (ICBN) nerves may play a role in the perception of tourniquet pain [19,49]. The likelihood of achieving MCBN block is greater with infraclavicular techniques. The ICBN is not blocked in either an ABPB or ICB [49]. To decrease tourniquet pain, raising a skin wheal on the medial aspect of the arm close to the axilla can potentially block the branches of the ICBN, thereby lessening the likelihood of tourniquet pain being experienced in these dermatomes by the patient [50].

Until recently, the use of US guidance during RA procedures was precluded by the image quality, portability, and affordability of these machines. Due to advances in this technology, US has become ubiquitous in most hospitals and operating theaters. This change is reflected in the increasing use of US for guiding nerve blocks, allowing us to develop novel techniques and increase success rates. Recent studies have all abandoned NS-guided blocks. The PERi-operative uSE of UltraSound (PERSEUS-RA) group has recently published guidelines for the European Society of Anesthesia and Intensive Care (ESAIC) and suggested US guidance for SCB, ICB, and ABPB [51]. The ASRA also published an executive summary on using US in RA procedures. They advocated using US due to improved block characteristics and possible efficiency improvement [52]. Even in low-income countries, US-guided RA continues to become more frequently available [53].

### 4.1. Limitations

Some limitations of this systematic review need to be addressed. First, the studies had inherent heterogeneity, as we included multiple techniques (US guidance, NS guidance, trans-arterial). Even within subgroups, different techniques of RA blocks (single-, double- or triple-injection techniques) were used, and mixtures of LA that were used varied (different concentrations, with or without additives). The volume of LA used per block also differed between a fixed dose and dosage per kilogram of body weight. Volumes of LA of ABPB varied widely, with the lowest being 20 mL and the highest being 60 mL. One study did not even define the injected volume but used a dose of 7 mg/kg lidocaine with epinephrine, considered the maximum safe dose for infiltration [21]. Of note, this heterogeneity, along with potential biases or variations in the methodologies, may influence the results of the meta-analyses. However, per study, the same LA mixtures were used, making the comparison in the RCTs between the investigated RA blocks adequate.

Second, many trials were conducted in single centers, and only two studies investigated over 100 participants per group [23,24]. The latter may impact the generalizability of our findings.

Third, the experience of the RA operators was not always described, which could affect performance time, failure rates, onset time, and patient satisfaction. Moreover, differences in sedation and pain medication during RA performance can have an impact on patient satisfaction and tourniquet pain.

Fourth, not all included RCTs offered a prospective registration code, and some were probably not registered [18,20,29,30,36,39]. Additionally, some discrepancies existed in some of the registered ones, for instance, differences in sample sizes [16,19].

Fifth, not all RA techniques have previously been studied. For IVRA, we only identified one RCT, and no studies concerning WALANT or distal, motor-sparing distal peripheral nerve blocks were found [1].

Sixth, the majority of surgical procedures involved wrist–hand surgeries. For this type of surgery, alternative techniques such as WALANT or selective distal, motor-sparing block of the median, ulnar, and/or radial nerves might also be possible and even preferred to assess range-of-motion intra-operatively.

Sixth, there are many techniques to perform the studied blocks, going beyond the use of US guidance as well as different dosing for a given block technique. This might impact the intra-group reproducibility and, in the end, the reported success rates. However, the pooled data in our meta-analysis represent a standardized mean difference between these groups.

Last, 42 procedures included in this systematic review studied elbow surgery as innervation of the medial side of the upper arm up to the elbow is supplied by the ICBN (T1-3) and the MCBN of the arm (C8-T1) [54,55]. These nerves are not blocked in either technique unless targeted separately.

### 4.2. Interpretation

ABPB is a safe approach to the brachial plexus with a relatively high surgical success rate and a fast onset time. Future investigations should focus on learning curve factors to assess whether new trainees could quickly learn ABPB in keeping with safety, time benefits, and even further improvement of surgical anesthesia success rates. Future research should focus on identifying the technique and dose with the highest success and lowest complication rates, after which, more robust studies can compare the different approaches to the brachial plexus (including WALANT, distal peripheral nerve blocks, and IVRA). Ideally, more robust RCTs with larger sample sizes should be performed.

## 5. Conclusions

US-guided ABPB had a surgical success rate at 30 min that was statistically lower compared to US-guided ICB and marginally lower compared to US-guided SCB. However, it had a better safety profile than other brachial plexus techniques and may be preferred in patients with significant comorbidities. Given the high heterogeneity of data and limited group sizes, care must be taken to generalize our findings to the whole population of patients undergoing brachial plexus block. Further research is needed to better understand the impact of different brachial plexus approaches on outcome parameters. In the meantime, the choice of technique should be individualized based on patient-related factors and the personal experience of the practitioner. In the case of an inadequate RA block, additional peripheral nerve blocks can be valuable rescue techniques.

## Figures and Tables

**Table 1:** Study characteristics. ABPB: axillary brachial plexus block, ICB: infraclavicular block, SCB: supraclavicular block, CB: coracoid block, MHB: mid-humeral block, NOS: not otherwise specified, NA: not available, US-guided: ultrasound-guided, NS-guided: nerve stimulator-guided.

	Author	Type of ABPB	First Intervention	Second Intervention	Group Numbers	Mean Age (years)	Male/Female Ratio	Surgery (Elbow/Forearm/ Wrist–Hand)
1	Tran et al., 2009 [15]	US-guided ABPB	US-guided ICB	US-guided SCB	40/40/40	51/42/40	71/49	11/34/75
2	Frederiksen et al., 2010 [36]	US-guided ABPB	US-guided ICB		40/40	50/50	36/44	4/21/55
3	Song et al., 2011 [18]	US-guided ABPB	US-guided ICB		11/11	49.5/37.9	16/6	0/22/0
4	López-Morales et al., 2013 [27]	US-guided ABPB	US-guided ICB		40/42	58.7/52.9	32/50	15/11/56
5	Boivin et al., 2016 [23]	US-guided ABPB	US-guided ICB		112/112	48/52	145/79	11/6/207
6	Stav et al., 2016 [16]	US-guided ABPB	US-guided ICB	US-guided SCB	34/23/37	60/63/63	48/46	NA
7	Vazin et al., 2016 [17]	US-guided ABPB	US-guided ICB	US-guided SCB	40/40/40	60/52/59	NA	0/30/90
8	Cemaloglu et al., 2018 [38]	US-guided ABPB	US-guided ICB		33/33	NA	NA	NA
9	Brenner et al., 2019 [19]	US-guided ABPB	US-guided ICB		40/42	51.9/54.5	33/49	0/70/11
10	Tran et al., 2008 [39]	NS-guided ABPB	US-guided ICB		35/35	46/50	47/23	1/27/42
11	Tedore et al., 2009 [24]	Trans-arterial ABPB	US-guided ICB		109/111	51/49	110/110	NA
12	Kapral et al. 1999 [25]	NS-guided ABPB	NS-guided ICB		20/20	48/46	22/18	NA
13	Deleuze et al., 2003 [31]	NS-guided ABPB	NS-guided ICB		50/50	45/47	56/44	NA
14	Ertug et al., 2005 [28]	NS-guided ABPB	NS-guided ICB		15/15	38.1/27	NA	NA
15	Koscielniak-N et al., 2005 [37]	NS-guided ABPB	NS-guided ICB		40/40	45/49	48/32	NA
16	Rettig et al., 2005 [32]	NS-guided ABPB	NS-guided ICB		30/30	45/59	26/34	NA/NA/26
17	Lahori et al., 2011 [40]	NS-guided ABPB	NS-guided ICB		30/30	NA	NA	NA
18	Kapral et al. 1994 [29]	US-guided ABPB	US-guided SCB		20/20	NA	NA	NA
19	Karmakar et al., 2012 [33]	US-guided ABPB	US-guided SCB		15/16	NA	NA	-/-/31
20	Arnuntasupakul et al., 2015 [20]	US-guided ABPB	US-guided SCB		20/20	45.6/42.6	20/20	-/3/37
21	Hussien et al., 2018 [30]	US-guided ABPB	US-guided SCB		40/40	42.7/45.5	41/39	-/-/80
22	Singh et al., 2010 [21]	NS-guided ABPB	NS-guided SCB	ABPB + SCB	25/25/25	33.5/35.9/30.8	60/15	NA
23	Fleck et al. 1994 [26]	Paraesthesia ABPB	NS-guided SCB		20/20	43.4/51.8	33/7	NA
24	Dardon et al., 2000 [41]	ABPB (NOS)	SCB (NOS)	CE	26/20/30	29/30/28	44/32	NA
25	Koscielniak-N. et al., 2000 [35]	NS-guided ABPB	NS-guided CB		29/30	49/55	40/19	NA
26	Bouaziz et al. 1997 [22]	NS-guided ABPB	NS-guided MHB		28/32	42/48	-	0/8/52
27	Fuzier et al., 2006 [34]	NS-guided ABPB	NS-guided MHB		45/45	36/40	65/25	0/5/85
28	Teunkens et al., 2020 [1]	US-guided ABPB	IVRA		60/60	50/53	57/63	0/0/120

ABPB: axillary brachial plexus block, ICB: infraclavicular block, SCB: supraclavicular block, CB: coracoid block, MHB: mid-humeral block, NOS: not otherwise specified, NA: not available, US-guided: ultrasound-guided, NS-guided: nerve stimulator-guided.

## Data Availability

The original data presented in the study are openly available in the supplements of this article.

## Supplementary Materials

The following supporting information can be downloaded at: https://www.mdpi.com/article/10.3390/jcm13113185/s1.
