# Supplementary material for: Axillary Brachial Plexus Block Compared with Other Regional Anesthesia Techniques in Distal Upper Limb Surgery: A Systematic Review and Meta-Analysis
Source: J Clin Med. 2024 May 29;13(11):3185. doi: 10.3390/jcm13113185 (PMC11173314; doi:10.3390/jcm13113185)

Appendix S1 Search strategies in the databases

MEDLINE search strategy

1. (((anesth* OR anaesth* OR analg*) ADJ3 regional) OR ((nerve OR plexus OR brachial) AND (block* OR anesth* OR anaesth* OR analg*))) OR (exp Brachial Plexus/ AND (exp Anesthesia, Conduction/OR exp Analgesia/))

2. ((axillar*) AND (interscalene OR supraclav* OR infraclav* OR brachial OR humeral OR elbow OR forearm OR bier OR intravenous OR WALANT)

3. 1 and 2

EMBASE search strategy

1. (((an?esth* OR analg*) AND regional) OR ((nerve OR plexus OR brachial) AND (block* OR an?esthe* OR analg*))):kw,ti,ab OR 'regional anesthesia'/exp

2. ((axillar*) and (interscalene OR supraclav* OR infraclav* OR brachial OR humeral OR elbow OR forearm OR Bier OR intravenous OR WALANT):kw,ti,ab

3. 1 and 2

CENTRAL search strategy

#1 brachial plexus[MeSH Terms]

#2 anesthesia[MeSH Terms]

#3 analgesia[MeSH Terms]

#4 (#1 AND (#2 OR #3))

#5 ((an?esth* OR analg*) near regional) OR ((nerve OR plexus OR brachial) AND (block* OR an?esthe* OR analg*))

#6 (#4 OR #5)

#7 (axillar*) AND (interscalene OR supraclavicul* OR infraclavicul* OR brachial OR humeral OR elbow OR forearm OR Bier OR Intravenous OR WALANT)

#8 (#6 AND #7)

Appendix S2 Risk of bias tool version 2 (ROB 2) [19]

	D1	D2	D3	D4	D5	Overall
1. Tran et al. 2009	+	+	+	+	+	+
2. Frederiksen et al. 2010	+	+	+	+	+	+
3. Song et al. 2011	+	+	+	!	+	!
4. López-Morales et al. 2013	+	+	+	!	+	!
5. Boivin et al. 2016	+	+	+	+	+	+
6. Stav et al. 2016	+	+	+	+	+	+
7. Vazin et al. 2016	+	+	+	+	+	+
8. Cemaloglu et al. 2018	!	-	+	-	!	-
9. Brenner et al. 2019	+	-	+	+	+	-
10. Tran et al. 2008	!	+	+	+	+	!
11. Tedore et al. 2009	+	+	+	!	+	!
12. Kapral et al. 1999	!	+	+	!	+	!
13. Deleuze et al. 2003	!	-	+	-	+	-
14. Ertug et al. 2005	!	-	!	+	+	-
15. Koscielniak-Nielsen et al. 2005	+	+	+	+	+	+
16. Rettig et al. 2005	+	+	+	+	+	+
17. Lahori et al. 2011	+	+	+	!	+	!
18. Kapral et al. 1994	!	+	+	-	+	-
19. Karmakar et al. 2012	!	!	+	-	+	-
20. Arnuntasupakul et al. 2015	+	+	+	+	+	+
21. Hussien et al. 2018	+	+	+	+	+	+
22. Singh et al. 2010	+	+	+	+	+	+
23. Fleck et al. 1994	!	-	+	-	+	-
24. Dardon et al. 2000	!	-	+	-	+	-
25. Koscielniak-Nielsen et al. 2000	+	+	+	+	+	+
26. Bouaziz et al. 1997	!	+	+	+	+	!
27. Fuzier et al. 2006	!	+	+	-	+	-
28. Teunkens et al. 2020	+	+	+	!	+	!

+ Low risk
! Some concerns
- High risk

D1 Randomisation process
D2 Deviations from the intended interventions
D3 Missing outcome data
D4 Measurement of the outcome
D5 Selection of the reported result

Appendix S3 Methodological quality graph: review authors' judgements about each methodological quality item presented as percentages across all included studies.

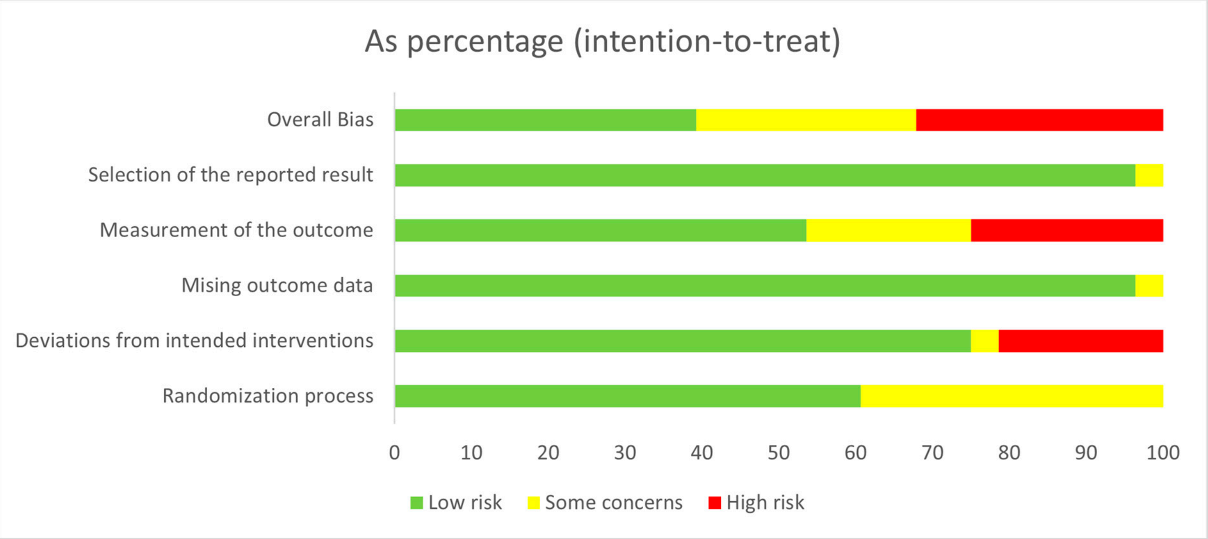

Appendix S4 Study characteristics on sedatives

	Author	Block sedation	Surgical sedation
1	Tran et al. 2009	Yes	Allowed
2	Frederiksen et al. 2010	Allowed	Allowed
3	Song et al. 2011	Not allowed	Not allowed
4	López-Morales et al. 2013	Yes	Allowed
5	Boivin et al. 2016	Allowed	Allowed
6	Stav et al. 2016	Yes	Not allowed
7	Vazin et al. 2016	Allowed	Not allowed
8	Cemaloglu et al. 2018	NA	NA
9	Brenner et al. 2019	Allowed	Allowed
10	Tran et al. 2008	Allowed	Allowed
11	Tedore et al. 2009	Allowed	Not allowed
12	Kapral et al. 1999	NA	NA
13	Deleuze et al. 2003	Yes	NA
14	Ertug et al. 2005	Yes	NA
15	Koscielniak-N et al. 2005	Yes	NA
16	Rettig et al. 2005	Yes	NA
17	Lahori et al. 2011	NA	Allowed
18	Kapral et al. 1994	Yes	Not allowed
19	Karmakar et al. 2012	NA	NA
20	Arnuntasupakul et al. 2015	Yes	Allowed
21	Hussien et al. 2018	Yes	NA
22	Singh et al. 2010	Yes	Not allowed
23	Fleck et al. 1994	Not allowed	Allowed
24	Dardon et al. 2000	Yes	NA
25	Koscielniak-N. et al. 2000	Yes	Allowed
26	Bouaziz et al. 1997	Yes	NA
27	Fuzier et al. 2006	Not allowed	Not allowed
28	Teunkens et al. 2020	Yes	Not allowed

NA: Not available

Appendix S5 Local anesthetics and volumes used in regional anesthesia blocks

	Author	Medication LA used	Volume LA
1	Tran et al. 2009	Lidocaine 1,5% + Epinephrine 1:200 000	35ml
2	Frederiksen et al. 2010	1/1 ratio Ropivacaine 0,75% + Mepivacaine 2% + Epinephrine 1:200 000	0,5ml/kg
3	Song et al. 2011	Lidocaine 5% + Epinephrine 1:200 000 + 0,1 mEq/ml NaBicarbonate	20ml
4	López-Morales et al. 2013	Levobupivacaine 0,5%	15-20ml
5	Boivin et al. 2016	Mepivacaine 1,5%	30ml
6	Stav et al. 2016	Bupivacaine 0,5% + Epinephrine 1:200 000	40ml
7	Vazin et al. 2016	Ropivacaine 0,75%	20ml
8	Cemaloglu et al. 2018	4/1 ratio Bupivacaine 0,5% + Lidocaine 2%	30ml ABPB - 25ml ICB
9	Brenner et al. 2019	Lidocaine 2% + Epinephrine 1:200 000	20-30ml
10	Tran et al. 2008	1/1 ratio Bupivacaine 0,5% + Lidocaine 2% + Epinephrine 1:200 000	35ml
11	Tedore et al. 2009	Mepivacaine 1,5% + Epinephrine 1:200 000 + 0,1mEq/ml NaBicarbonate	40-60ml based on BW
12	Kapral et al. 1999	Mepivacaine 1%	40ml
13	Deleuze et al. 2003	Ropivacaine 0,75%	40ml
14	Ertug et al. 2005	Bupivacaine 0,375%	40ml
15	Koscielniak-N et al. 2005	1/1 ratio Ropivacaine 0,75% + Mepivacaine 2% + Epinephrine 1:200 000	0,5ml/kg
16	Rettig et al. 2005	Ropivacaine 0,75%	0,5ml/kg
17	Lahori et al. 2011	1/2 ratio Bupivacaine 0,5% + Lidocaine 2%	40ml
18	Kapral et al. 1994	Bupivacaine 0,5%	30ml
19	Karmakar et al. 2012	Ropivacaine 0,5%	20ml
20	Arnuntasupakul et al. 2015	Lidocaine 1,5% + Epinephrine 1:200 000	29ml ABPB - 32ml SCB
21	Hussien et al. 2018	Bupivacaine 0,5%	35ml ABPB - 25ml SCB
22	Singh et al. 2010	Lidocaine + Ephinephrine	7mg/kg
23	Fleck et al. 1994	Mepivacaine 1,5%	50ml
24	Dardon et al. 2000	1/4 ratio Bupivacaine 0,5% + Lidocaine 2%	40ml ABPB/SCB - 10ml epidural
25	Koscielniak-N. et al. 2000	Ropivacaine 0,75%	20-40ml based on BW
26	Bouaziz et al. 1997	Lidocaine 1,5% + Epinephrine 1:200 000	40ml
27	Fuzier et al. 2006	Ropivacaine 0,75%	40ml
28	Teunkens et al. 2020	Mepivacaine 1% ABPB - Lidocaine 0,75% IVRA	28ml ABPB - 40ml IVRA

ABPB: Axillary brachial plexus block, ICB: Infraclavicular block, SCB: Supraclavicular block, BW: body weigh

Appendix S6 Forest plot of block performance time in minutes NS technique

Study or Subgroup	NS-guided ABPB Mean	SD	Total	NS-guided ICB Mean	SD	Total	Weight	Std. Mean Difference IV, Random, 95% CI
Deleuze et al. 2003	6	2.8	50	2.5	1.9	50	33.6%	1.45 [1.01, 1.89]
Koscielniak-Nielsen et al. 2005	7	2	40	4	1	40	33.3%	1.88 [1.35, 2.41]
Rettig et al. 2005	5.3	1.8	30	9.2	2.7	30	33.1%	-1.68 [-2.27, -1.08]
**Total (95% CI)**			**120**			**120**	**100.0%**	**0.56 [-1.46, 2.58]**

Heterogeneity: Tau² = 3.12; Chi² = 91.12, df = 2 (P < 0.00001); I² = 98%
Test for overall effect: Z = 0.54 (P = 0.59)

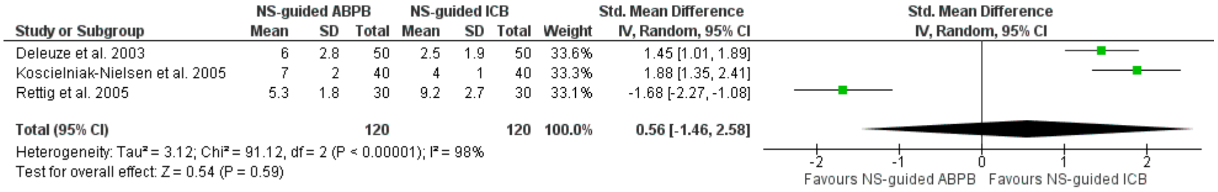

Appendix S7 Complications after regional anesthesia in the included studies

	Author	Pneumothorax	Vascular puncture	Horner's syndrome	LAST	Neuropraxia
1	Tran et al. 2009		2.5% US-guided ABPB<br>2.5% US-guided SCB<br>2.5% US-guided ICB	0% US-guided ABPB<br>37,5% US-guided SCB<br>5% US-guided ICB	0%	
2	Frederiksen et al. 2010	0%	5% US-guided ABPB<br>7.5% US-guided ICB			
4	López-Morales et al. 2013		5% US-guided ABPB<br>2.3% US-guided ICB			
5	Boivin et al. 2016	0%	0.89% US-guided ABPB<br>1.78% US-guided ICB		0%	0%
6	Stav et al. 2016	0%	0%	0% US-guided ABPB<br>8.10% US-guided SCB<br>0% US-guided ICB	0%	0%
7	Vazin et al. 2016	0%	0%	0%	0%	0%
9	Brenner et al. 2019		8.33% US-guided ABPB<br>11.43% US-guided ICB			
10	Tran et al. 2008		25.71% NS-guided ABPB<br>5.71% US-guided ICB	0% US-guided ABPB<br>2.86% US IC-guided IC	2,86% US-guided ABPB<br>2.86% US-guided ICB	
11	Tedore et al. 2009					3% transarterial ABPB<br>2% US-guided ICB
12	Kapral et al. 1999	0%	10% NS-guided ABPB<br>0% NS-guided ICB			
13	Deleuze et al. 2003	0%	0% NS-guided ABPB<br>4% NS-guided ICB	0% NS-guided ABPB<br>4% NS-guided ICB		0%
15	Koscielniak-N et al. 2005	0%	0% NS-guided ABPB<br>16.67% NS-guided ICB	0%		0%
16	Rettig et al. 2005	0%		0% NS-guided ABPB<br>3% NS-guided ICB		
17	Lahori et al. 2011	0%	30% NS-guided ABPB<br>6.67% NS-guided ICB	0%		
18	Kapral et al. 1994	0%	0%	0%		0%
20	Arnuntasupakul et al. 2015		0%	0% US-guided ABPB<br>45% US-guided SCB		
21	Hussien et al. 2018	0% US-guided AE<br>10% US-guided S	2.5% US-guided ABPB<br>2.5% US-guided SCB	0% US-guided ABPB<br>32.5% US-guided SCB		15% US-guided ABPB<br>10% US-guided SCB
22	Singh et al. 2010	0%	0%	0%		0%
23	Fleck et al. 1994	0%	0%	0%		
25	Koscielniak-N. et al. 2000		6.9% NS-guided ABPB			0%
28	Teunkens et al. 2020				0% US-guided ABPB<br>3.3% IVRA	

ABPB: Axillary brachial plexus block, ICB: Infraclavicular block, SCB: Supraclavicular block

Appendix S8 Comparison of complications: Pneumothorax US-guided ABPB vs other blocks

Study or Subgroup	US-guided ABPB Events	US-guided ABPB Total	Other blocks Events	Other blocks Total	Weight	Risk Ratio M-H, Random, 95% CI
Boivin et al. 2016	0	112	0	112		Not estimable
Frederiksen et al. 2010	0	40	0	40		Not estimable
Hussien et al. 2018	0	40	4	40	100.0%	0.11 [0.01, 2.00]
Kapral et al. 1994	0	20	0	20		Not estimable
Stav et al. 2016	0	34	0	60		Not estimable
Vazin et al. 2016	0	40	0	80		Not estimable
**Total (95% CI)**		**286**		**352**	**100.0%**	**0.11 [0.01, 2.00]**
Total events	0		4			

Heterogeneity: Not applicable
Test for overall effect: Z = 1.49 (P = 0.14)

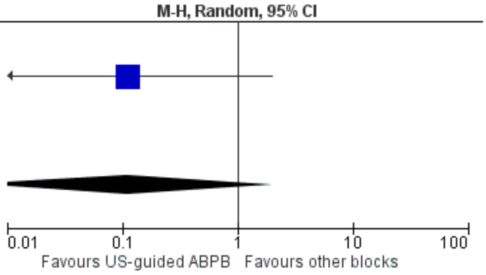

Appendix S9 Comparison of complications: Vascular puncture US-guided ABPB vs other blocks

Study or Subgroup	US-guided ABPB Events	US-guided ABPB Total	Other blocks Events	Other blocks Total	Weight	Risk Ratio M-H, Random, 95% CI
Arnuntasupakul et al. 2015	0	20	0	20		Not estimable
Boivin et al. 2016	1	112	2	112	10.8%	0.50 [0.05, 5.44]
Brenner et al. 2019	3	40	4	42	29.9%	0.79 [0.19, 3.30]
Frederiksen et al. 2010	2	40	3	40	20.4%	0.67 [0.12, 3.78]
Hussien et al. 2018	2	40	2	40	16.8%	1.00 [0.15, 6.76]
Kapral et al. 1994	0	20	0	20		Not estimable
López-Morales et al. 2013	2	40	1	42	11.0%	2.10 [0.20, 22.26]
Stav et al. 2016	0	34	0	60		Not estimable
Tran et al. 2009	1	40	2	80	10.9%	1.00 [0.09, 10.70]
Vazin et al. 2016	0	40	0	80		Not estimable
**Total (95% CI)**		**426**		**536**	**100.0%**	**0.86 [0.39, 1.89]**
Total events	11		14			

Heterogeneity: Tau² = 0.00; Chi² = 0.88, df = 5 (P = 0.97); I² = 0%
Test for overall effect: Z = 0.37 (P = 0.71)

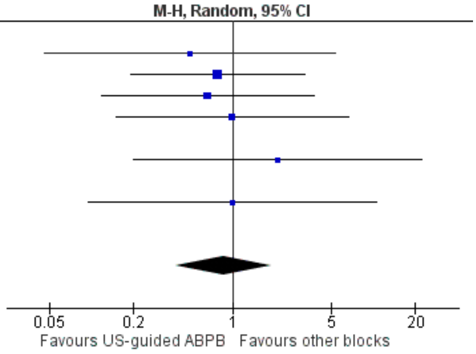

Appendix S10 Comparison of complications: Horner's syndrome US-guided ABPB vs US-guided SCB

Study or Subgroup	US-guided ABPB Events	US-guided ABPB Total	US-guided SCB Events	US-guided SCB Total	Weight	Risk Ratio M-H, Random, 95% CI
Arnuntasupakul et al. 2015	0	20	9	20	50.2%	0.05 [0.00, 0.85]
Hussien et al. 2018	0	40	13	40	49.8%	0.04 [0.00, 0.60]
Kapral et al. 1994	0	20	0	20		Not estimable
**Total (95% CI)**		**80**		**80**	**100.0%**	**0.04 [0.01, 0.32]**
Total events	0		22			

Heterogeneity: Tau² = 0.00; Chi² = 0.03, df = 1 (P = 0.86); I² = 0%
Test for overall effect: Z = 3.11 (P = 0.002)

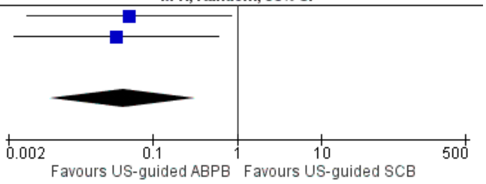

Supplement: Supplementary file 1 [file jcm-13-03185-s001.zip › Supplementary document.pdf]
